# Giant photostriction in organic–inorganic lead halide perovskites

**DOI:** 10.1038/ncomms11193

**Published:** 2016-04-05

**Authors:** Yang Zhou, Lu You, Shiwei Wang, Zhiliang Ku, Hongjin Fan, Daniel Schmidt, Andrivo Rusydi, Lei Chang, Le Wang, Peng Ren, Liufang Chen, Guoliang Yuan, Lang Chen, Junling Wang

**Affiliations:** 1 School of Materials Science and Engineering, Nanyang Technological University, Block N4.1-02-24, 50 Nanyang Avenue, Singapore 639798, Singapore; 2 School of Physical and Mathematical Sciences, Nanyang Technological University, Singapore 639798, Singapore; 3 Singapore Synchrotron Light Source, National University of Singapore, 5 Research Link, Singapore 117603, Singapore; 4 Department of Materials Science and Engineering, Nanjing University of Science and Technology, Nanjing 210094, China; 5 Department of Physics, South University of Science and Technology of China, Shenzhen 518055, China

## Abstract

Among the many materials investigated for next-generation photovoltaic cells, organic–inorganic lead halide perovskites have demonstrated great potential thanks to their high power conversion efficiency and solution processability. Within a short period of about 5 years, the efficiency of solar cells based on these materials has increased dramatically from 3.8 to over 20%. Despite the tremendous progress in device performance, much less is known about the underlying photophysics involving charge–orbital–lattice interactions and the role of the organic molecules in this hybrid material remains poorly understood. Here, we report a giant photostrictive response, that is, light-induced lattice change, of >1,200 p.p.m. in methylammonium lead iodide, which could be the key to understand its superior optical properties. The strong photon-lattice coupling also opens up the possibility of employing these materials in wireless opto-mechanical devices.

## Abstract (web summary)

The photophysics of lead halide perovskites is under intense investigation. Here, the authors use force microscopy on single crystals to show that light induces drastic lattice changes, and propose that the weakening of the hydrogen coupling under illumination is responsible for the lattice dilatation.

## Graphical abstract

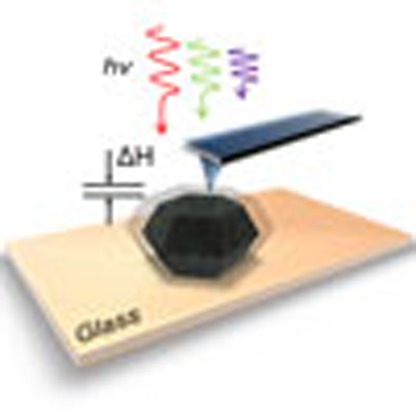

The past few years witnessed the explosion of research on photovoltaic cells based on the hybrid organic–inorganic perovskites^1^,^2^,^3^,^4^,^5^, in particular methylammonium lead iodide (MAPbI_3_). Concomitantly, these materials have also been explored for lasers^6^, light emitting diodes^7^ and photodetectors^8^. Besides improving the photovoltaic cell efficiency, much work has been devoted to the mechanism behind their extraordinary performances. Anomalously long lifetime and diffusion length of photo-carriers have been observed and related to the high efficiency^9^,^10^,^11^,^12^. In solution processed thin films of mixed halide perovskites, carrier lifetime of longer than 1 μs^13^ and diffusion length exceeding 1 μm^11^ were reported. These values can be further increased in high-quality MAPbI_3_ single crystals with greatly suppressed trap-state densities^14^,^15^, suggesting an even higher attainable efficiency approaching the Shockley–Queisser limit^16^. However, to become commercially viable, the long-term stability of these materials has to be significantly improved. Despite the intensive research efforts, the origin of the long-carrier lifetime and diffusion length remains elusive. The bi-molecular recombination rate deviates from Langevin theory by orders of magnitude, which underlines the unique attribute of hybrid perovskites compared with conventional low-mobility semiconductors, but is reminiscent of disordered material systems such as amorphous Si and organic solar cells^17^,^18^. These clues lead us to ponder on the role of the organic molecules on the material's unusual photophysical properties. These molecules are dynamically tumbling inside the inorganic scaffold due to the small rotational energy barrier^19^,^20^. Even though the organic group is not directly involved in the electronic structure around the band edges, it may interact with the inorganic PbI_6_ octahedron through its rotational degree of freedom, as revealed in recent density functional theory (DFT) calculations^21^,^22^. Experimentally, hydrogen bonding between the halides and the amine group at room temperature was confirmed by infrared spectroscopy^23^, supporting the theoretical results.

Here we provide yet another evidence for the interaction between the organic and inorganic moieties in the hybrid perovskites. A giant photostrictive response, namely, light-induced lattice change, of >1,200 p.p.m. was observed in MAPbI_3_. Careful analysis suggests that the strong photon-lattice coupling may arise from the weakening of the hydrogen bonding between N and I by the photo-generated carriers. Not only could it shed light on the anomalous photophysics, this discovery also opens up new possibilities for these fascinating materials, enabling novel device paradigms such as photo-driven microsensing and microactuation^24^,^25^.

## Results

### Photostriction in MAPbI_3_ single crystals

**Figure 1:**
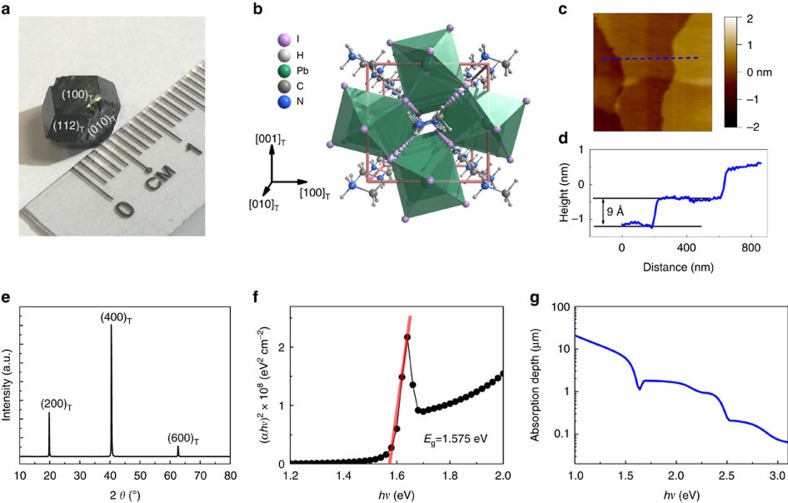
Basic properties of the MAPbI_3_ single crystals. (**a**) Photograph of a MAPbI_3_ single crystal used in this study. (**b**) Perspective view of the unit cell of tetragonal MAPbI_3_. (**c**) Typical topography of the single crystal obtained by AFM, showing smooth surface with unit-cell steps. The scan size is 1 × 1 μm. (**d**) Height profile along the blue dash line denoted in **c**. (**e**) X-ray diffraction pattern of the crystal, which only shows tetragonal (*l*00) type peaks, indicating the high quality of the single crystal. (**f**) Direct-transition tauc plot according to the absorption coefficient of the crystal, from which a direct band gap of 1.575 eV is extracted. The red solid line is the linear fit. (**g**) The penetration depth deduced from the absorption coefficient.

MAPbI_3_ single crystals as large as 10 × 8 × 8 mm are prepared using solution growth method. Photograph of a typical sample with well-defined facets used in this study is shown in Fig. 1a. Only those peaks corresponding to the tetragonal (*l*00) planes (Fig. 1b) are present in the X-ray diffraction scan (Fig. 1e), confirming the high quality of the single crystal. Based on the absorption coefficient deduced from the spectroscopic ellipsometry (Supplementary Fig. 1), an abrupt excitonic absorption at 1.575 eV has been determined (Fig. 1f), which is consistent with previous reports^14^,^15^. The topography and photo-induced dimension change are investigated using an atomic force microscope (AFM) over the atomically flat surface of the sample as shown in Fig. 1c,d.

**Figure 2:**
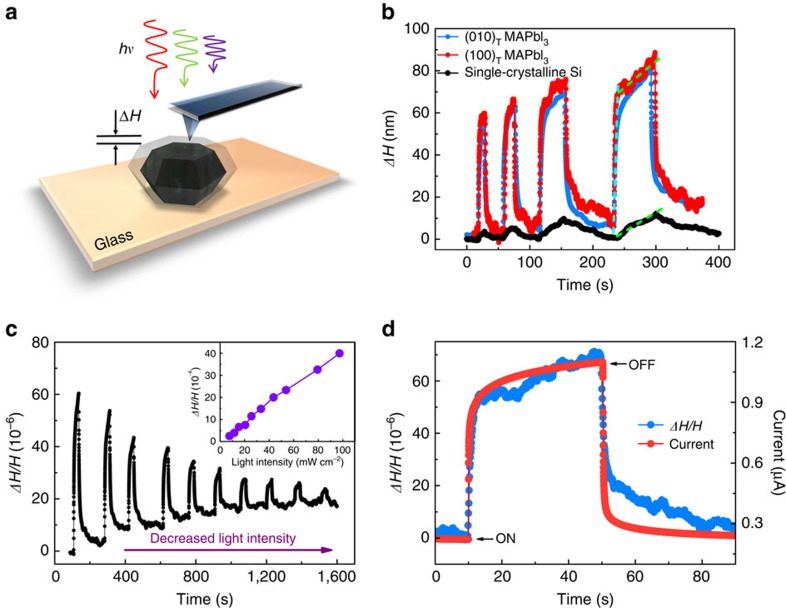
Photostrictive effect of MAPbI_3_ single crystals. (**a**) Schematic drawing of the experimental set-up for the photostrictive measurements. The AFM tip is fixed at one point on the sample to record the height as a function of time. (**b**) Photostriction in the MAPbI_3_ single crystal. Both (100)_T_- and (010)_T_-oriented single crystals produce a similar elongation of ∼50 p.p.m. under 100 mW cm^−2^ white-light illumination. The height change of a Si single crystal is also measured as a comparison. Cyan and green dash lines delineate the fast and slow components of the height change, respectively. (**c**) Light intensity dependence of the photostrictive effect. The inset shows the proportional relationship between the photostriction and light intensity. (**d**) A comparison between the height and current changes on light irradiation.

Interestingly, when light is shining on the crystal, a sudden change in the dimension is observed. Figure 2a schematically depicts the experimental set-up for the measurements. Single crystals with various facets facing up are glued on the glass substrates using silver paint. The crystals are then illuminated from top surface using a halogen lamp with a continuous spectrum ranging from 400 to 750 nm (Supplementary Fig. 2). The light goes through the AFM built-in optical system onto the surface of the sample. By placing the AFM tip at the surface, the sample height is recorded as a function of time and illumination conditions (see Methods). As shown in Fig. 2b, under 100 mW cm^−2^ white-light illumination, a reproducible change in the sample height is clearly observed. Both (100)_T_- and (010)_T_-oriented (subscript T denotes tetragonal index) single crystals produce a similar elongation of approximately 50 nm along the vertical direction. Considering the crystal thickness of approximately 1 mm, this translates into a photostriction (defined as the height change divided by the sample thickness *ΔH/H*) of 5 × 10^−5^ (or 50 p.p.m.). To confirm that the observed height change is an intrinsic material property instead of a light-induced measurement artifact, we also tested Si and SrTiO_3_ (STO) single crystals (Fig. 2b and Supplementary Fig. 3a), both of which should show negligible photostrictive effects. Instead of a sudden change of height, both samples exhibit a much slower response, whose magnitude scales with the illumination time. This suggests a possible thermal effect. However, the very different optical absorption and thermal expansion coefficients of Si and STO are at odds with the similar responses. Furthermore, this slow response can also be seen for MAPbI_3_ as indicated in Fig. 2b. Thus, we infer that this material-independent response is a measurement artifact that results from the heating-induced bending of the AFM tip rather than the samples under test. To exclude the possible contribution from the thermal expansion of the sample, the temperature of the sample surface was monitored under the same illumination conditions (Supplementary Fig. 3b). Clearly, the temperature profile does not match the sudden height change. Besides, a brief estimation based on the material parameters of MAPbI_3_ gives a thermal expansion on the order of 0.1 nm (Supplementary Note 1), much smaller than the response observed.

Further investigation shows that the photostrictive response is proportional to the light intensity (Fig. 2c), and no saturation is observed up to 100 mW cm^−2^, which suggests a possible correlation between the photostriction and photo-generated carriers. To check the photoconductivity of the sample, we have measured the current under the same illumination condition by applying 1 V bias to the Pt electrodes coated on the two opposite facets of the crystal. As shown in Fig. 2d, the sample height change follows the same profile as the current change on illumination. Since the conductivity is proportional to the amount of free carriers (provided there is no significant change in the carrier mobilities), it implies that the photostriction is directly related to the photo-generated carriers, that is, of an electronic origin. The long tail of the height signal, after the light is turned off, is attributed to the slow thermal relaxation of the AFM tip. This is supported by the fact that it scales with the illumination time (Fig. 2b).

### Photon energy dependence

**Figure 3:**
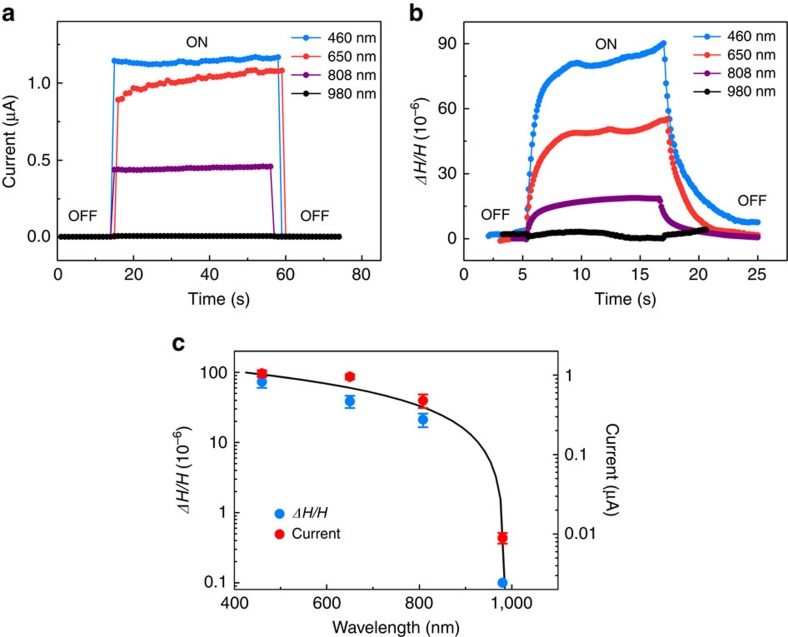
Photon energy dependence of the photostriction. (**a**) When the photon energy is close to or above the band gap (that is, 460, 650 and 808 nm lasers), large current on illumination (under 1 V voltage) is observed, whereas negligible photocurrent is obtained when photon energy is below the band gap (that is, 980 nm laser). (**b**) The photostriction shows similar behaviour to that of photocurrent. (**c**) Photocurrents and height changes of the single crystal as functions of the incident photon energy, showing clear correlation between these two properties. The light intensity was kept at ∼100 mW cm^−2^ for all lasers. The black solid line serves as a guide to the eye.

If the photo-generated free carriers are indeed responsible for the dimension change, it must depend on the incident photon energy. As revealed by the energy-dependent absorption coefficient (Fig. 1f), the excitonic absorption of MAPbI_3_ single crystal is 1.575 eV with the true band gap being a few tens of meV above that. When the lasers with photon energy higher than or close to the band gap of MAPbI_3_ are used (460, 650 and 808 nm), efficient generation of electron–hole pairs can be expected and thus large photoconductivity is obtained (Fig. 3a, the bias applied is 1 V). When 980 nm laser is used, no photocurrent is observed since its photon energy is below the band gap of MAPbI_3_. Similarly, the photostrictive response shows clear photon energy dependence as well (Fig. 3b). When we plot the photocurrent and photostriction together as functions of the incident photon energy (Fig. 3c), the correlation is even clearer. This observation again confirms that the photostrictive response in MAPbI_3_ single crystal is caused by photo-generated carriers.

### Giant photostriction in MAPbI_3_ film

**Figure 4:**
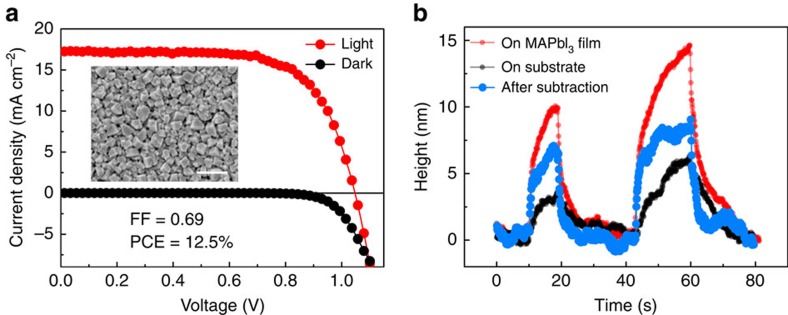
Photovoltaic and photostrictive properties of MAPbI_3_ thin films. (**a**) Typical current density–voltage characteristic of a MAPbI_3_ thin-film photovoltaic cell (FTO/TiO_2_/MAPbI_3_/Spiro-OMeTAD/Ag) under simulated AM1.5 100 mW cm^−2^ illumination (red line) and under dark (black line). The power conversion efficiency can reach 12.5%. The inset shows the SEM image of the MAPbI_3_ thin film. The scale bar, 1 μm. (**b**) Height change of the MAPbI_3_ thin film (4 μm) on FTO-coated glass substrate under illumination. The net response from the film can be obtained by substracting the extrinsic contribution from the substrate. Under 100 mW cm^−2^ white light, about 5 nm height change can be observed, corresponding to a photostriction of 1,250 p.p.m.

According to the Beer–Lambert law, the intensity of incident light decays exponentially into the material's bulk. The strong absorption of MAPbI_3_ in visible spectrum results in a penetration depth less than a few micrometres (Fig. 1g), leaving most of the crystal not illuminated and the effective photostriction (*ΔH/H*) likely underestimated. To address this issue, we have carried out thickness-dependent photostriction measurement on small flakes cleaved from the same single crystal (Supplementary Fig. 4). It is found that for a 700-μm-thick flake, the height change of 50 nm is comparable to those of crystals with thicknesses greater than or equal to 1 mm, suggesting a saturated value. However, for a 150-μm-thick flake, the photostrictive response dramatically reduces to about 20 nm. Note that this thickness is already smaller than the carrier diffusion length estimated for single crystals^15^. Hence, we argue that although the light absorption depth is only a few micrometres, the photo-excited carriers can diffuse deep into the bulk, leading to a much thicker responsive layer. This conclusion helps us to further separate the photostriction signal into a fast near-surface response (absorption limited), a relatively slower contribution from the bulk limited by diffusion of the photo-carriers, and finally the much slower plateau due to the thermal bending of the AFM tip (Supplementary Note 2). The calculated photostriction of the 150-μm-thick flake is greater than 100 p.p.m. Last, we prepare MAPbI_3_ thin films of 4 μm thick on fluorine-doped tin oxide (FTO)-coated glass and measure its photostrictive response. The quality of the film is confirmed by the performance of a testing cell (FTO/TiO_2_/MAPbI_3_/Spiro-OMeTAD/Ag), in which an efficiency of 12.5% is obtained (Fig. 4a). Since the slow height response is likely due to the thermal effect of the AFM tip, we subtract it from the height change profile and obtain a thickness change of about 5 nm under 100 mW cm^−2^ white light, which translates into a photostrictive response of approximately 1,250 p.p.m. in the MAPbI_3_ film (Fig. 4b). This is much larger than any of the reported intrinsic photostrictive effect in ferroelectric materials, polar and non-polar semiconductors^24^.

## Discussion

In the literature, photostriction has been reported for several non-polar semiconductors, polar materials (including ferroelectrics), chalcogenide glasses and organic materials. Following the work of Kundys^24^, we analyse the experimentally observed photostrictive coefficients (normalized to the light intensity) of known materials as shown in Table 1 (refs 24, 26, 27, 28, 29, 30, 31, 32, 33, 34, 35, 36, 37, 38, 39, 40). In non-polar semiconductors, it is related to the pressure susceptibility of the energy gap based on the deformation potential theory^41^. This is justified by the opposite signs of d*E*_g_/d*P* in Ge and Si and the corresponding photostrictive responses^26^,^27^. Recently, it is reported that the band gap of CH_3_NH_3_PbBr_3_ (MAPbBr_3_) reduces with increasing hydrostatic pressure^42^, which should lead to the contraction of the lattice under illumination, contrary to our results. Besides, this effect is usually small, inconsistent with the giant photostriction we observed. In polar materials, the photostriction is closely linked to the bulk photovoltaic effect and commonly interpreted as a consequence of the converse piezoelectric effect^24^. Owing to the non-centrosymmetric structure, photo-generated carriers are spontaneously separated to produce an effective electric field that deforms the lattice through the piezoelectric tensor. However, despite many theoretical and experimental studies advocating for the ferroelectricity in MAPbI_3_ (refs 43, 44, 45, 46), a strong proof remains lacking^47^. While the structure of the low-temperature orthorhombic phase was refined to be centrosymmetric^48^,^49^, the high-temperature structure is difficult to be determined due to the dynamic disorder of the CH_3_NH_3_^+^ group. Considering the dynamic motion of the molecular dipole at room temperature, a long-range ferroelectric order can hardly exist, as confirmed by our polarization measurements, as well as piezoelectric force microscopy (Supplementary Figs 5 and 6). Even if polar nanoregions exist in MAPbI_3_ as ferroelectric relaxors^50^, a completely random distribution of the electric dipoles should cancel out the lattice change along all directions because of the volume conservation during piezoelectric deformation. For chalcogenide glasses and organic materials, both of them exhibit huge photostrictive response, which can be attributed to photo-induced bond modifications or photoisomerizations^51^,^52^,^53^. Strictly speaking, they should be classified as photochemical effects, most of which are irreversible after the irradiation. Besides, their responses are usually very slow, on the order of minutes or even hours, though some exceptions are recently reported^40^,^54^. It thus appears that none of these mechanisms can account for our observation.

**Figure 5:**
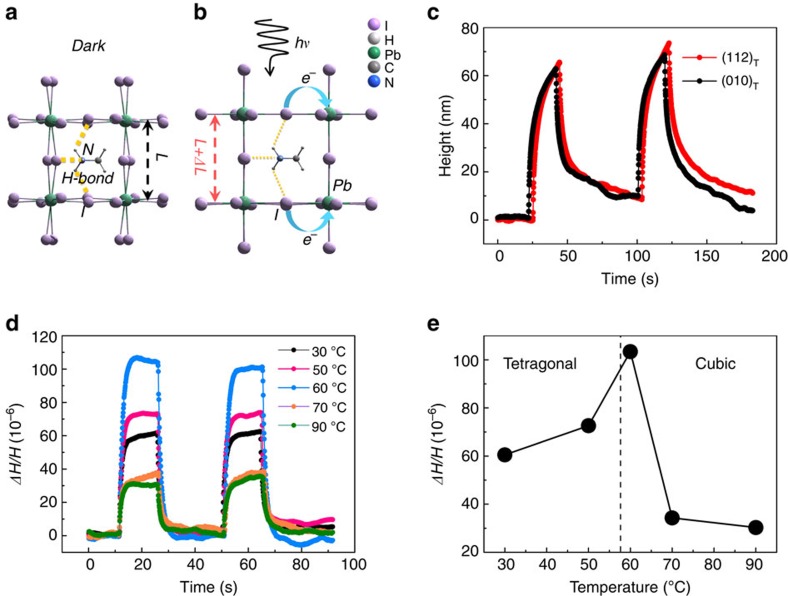
Proposed mechansim for the giant photostriction in MAPbI_3_. (**a**,**b**) Schematic illustrations (not to scale) showing that the weakening of the hydrogen bonding between the amine group and the iodine ion by photo-generated carriers leads to the lattice expansion. (**c**) Orientation dependence of photostriction of a MAPbI_3_ single crystal shows similar magntidue due to the isotropic expansion of the lattice. (**d**,**e**) Temperature-dependent photostriction of a MAPbI_3_ single crystal across the tetragonal-cubic phase transition under 100 mW cm^−2^ white light. The phase transition point is indicated by the black dash line. Enhanced lattice susceptibility around the phase transition boundary is likely responsible for the larger photostriction observed.

Furthermore, it should be noted that the possible contribution from thermal expansion has been ruled out, and only the intrinsic effect is discussed here. By comparing the values listed in Table 1, it is clear that the photostrictive coefficient of MAPbI_3_ happens to lie in between the inorganic and organic materials (chalcogenide glasses are an exception due to their amorphous nature), which is consistent with its hybrid character and mechanical properties^55^. The magnitude of the photostriction relies on how large a material's lattice can distort. In inorganic crystals, the atoms are closely packed with strong covalent or ionic bonds. Hence, it will be energetically costly for their lattices to deform. Organic materials, on the contrary, are at the other extreme. The small molecules or polymer chains are glued together by hydrogen bonds or van de Waals interactions, and their lattices can deform significantly due to the large intermolecular spacing. It suggests that the organic group may play a vital role in the giant photostriction effect observed in hybrid perovskites. In fact, hydrogen bonding between organic and inorganic moieties has long been studied in hybrid materials, which may lead to emergent properties^56^,^57^,^58^. Specifically in hybrid perovskites, hydrogen bonding between the amine group and the halide ions has been verified both theoretically and experimentally^23^,^59^,^60^. In this regard, we propose that it is the weakening of the hydrogen bonding by photo-generated carriers that results in the lattice dilation. As schematically shown in Fig. 5a, the hydrogen bonding between N and I is geometrically coupled to the buckling of the Pb–I–Pb bond and the tilting of the iodine octahedron. Under above-band-gap illumination, the first direct transition corresponds to the charge transfer from hybridized Pb *6s*–I *5p* orbital to the Pb *6p* orbital, forming weakly bound excitons that are easily dissociated by thermal energy^61^,^62^,^63^. The electronic transition directly leads to the reduction of electron density on the I site, and thereby reduces its Coulomb interaction with the amine group. This in turn straightens the Pb–I–Pb bond and results in a larger interatomic spacing (Fig. 5b).

If our analysis is correct, a couple of predictions can be made. First, the disordered orientations of the organic molecules, as well as the elastic multidomain of the crystal should lead to an isotropic photostrictive response, which is indeed observed in our orientation-dependent measurements shown in Fig. 5c. The result of the polycrystalline MAPbI_3_ film provides another piece of evidence. Second, as the hydrogen bonding is intimately coupled to the octahedral tilt, divergence in the photostrictive response is expected around the tetragonal-cubic phase transition. Once again, our temperature-dependent measurements confirm this prediction (Fig. 5d,e). The photostriction measured at 60 °C is almost twice of that measured at room temperature, which can be interpreted by the enhanced lattice susceptibility at the structure transition boundary. The photostriction is reduced in the high-temperature cubic phase, but still with appreciable magnitudes. As pointed out by Quarti *et al.*^64^, although the high-temperature phase appears cubic at a large scale, the local structure may strongly deviate from the nominal cubic one at any given time owing to the fluctuation of the organic molecule inside the inorganic framework. Thus, the interaction between them, though reduced, does not completely vanish.

The giant photostriction suggests strong photon-lattice coupling in MAPbI_3_. It has important implications for understanding the exceptional photovoltaic performance of hybrid perovskites. For instance, the lattice expansion and reduced Coulomb interaction between the organic group and the inorganic framework will further reduce the rotational barrier for the molecule dipole. The enhanced tumbling of the electric dipole may lead to a dynamic change of the local band structure that suppresses the electron–hole recombination^22^. This scenario is in line with the decrease of the bi-molecular recombination rate at high temperatures^65^. Besides being important to explain the long-carrier lifetime and diffusion length in the hybrid lead halide perovskites, the giant photostriction also opens up new pathways for applications in opto-mechanical devices^24^,^25^.

## Methods

### Growth of MAPbI_3_ single crystal and structure characterization

CH_3_NH_3_I was first prepared according to previous report^66^. Typically, 27.8 ml methylamine (40% in methanol, Aldrich) was added into 30 ml hydroiodic acid (57 wt.% in water, Aldrich) and stirred for 2 h at 0 °C. After removing the solvent by rotary evaporating at 50 °C, the product was washed with diethyl ether and then recrystallized with ethanol. White crystals were obtained and dried in a vacuum box. PbI_2_ (1.157 g, 99%, Aldrich) and the as prepared CH_3_NH_3_I (0.395 g) were mixed in 2 ml γ-butyrolactone at 60 °C with stirring. Then, 1 ml acetonitrile (Aldrich) was added into the solution and a clear pale yellow solution was obtained. The solution was then placed in a vial and kept in an oven at 70 °C for 20 min. Small MAPbI_3_ single crystals then appeared at the bottom of the vial. One of these small crystals was picked out and put into another vial with the same solution for continuous growth. The crystals used for measurement were grown for 3 h. X-ray diffraction data were collected on a high-resolution diffractometer (Bruker D8 Discover) using Cu K_α_ radiation.

### Thin-film solar cell testing

Photovoltaic cell with the structure of FTO/TiO_2_/MAPbI_3_/Spiro-OMeTAD/Ag was prepared to measure the power conversion efficiency. Under illumination of solar-simulated AM1.5 sunlight at 100 mW cm^−2^, the current–voltage curve was obtained using a pA meter/direct current (DC) voltage source (Hewlett Package 4140B).

### Spectroscopic ellipsometry

The spectroscopic ellipsometry measurements have been performed using a commercially available rotating analyser instrument with compensator (V-VASE; J.A. Woollam Co., Inc.) within the spectral range from 0.6 to 6 eV. Data has been collected at two incidence angles (50° and 70°), while the sample was continuously purged with nitrogen gas to avoid degradation. The absorption coefficient was then calculated from the pseudodielectric function (Supplementary Fig. 1).

### Atomic force microscopy and piezoelectric force microscopy

Two commercial AFMs were used to measure the photostrictive responses: Asylum Research (AR) MFP 3D and Park XE 150 under ambient condition (20–30% relative humidity, 25 °C). Although the surface of the crystal degrades slowly with time in ambient condition possibly due to surface hydration, it does not affect the photostrictive response significantly. We believe this is because the hydration product is an optically transparent layer, which does not affect the absorption and thus the photostriction of the bulk. During the photostriction test, the light (from either a halogen lamp or laser diodes) was guided through an optical fibre into the built-in optical microscope of the AFM. For the AR AFM system, the light has to go through its internal optics, which include a cold mirror that reflects only visible light. As such, only the white-light tests were carried out using AR AFM. The wavelength-dependent tests were performed on a Park AFM system, whose optics system includes only an optical microscope that allows all the studied wavelengths (460, 650, 808 and 980 nm) to pass through. In both systems, the light was shined from above the AFM tip. However, because the light had gone through an optical fibre, then been focused by the optical lens, the light was no more collimated. Therefore, the region right beneath the AFM tip was not shadowed (Supplementary Note 3). Furthermore, the beam size was about 0.1 cm^2^ for all light sources. The height profile as a function of time, with the light periodically irradiated on the surface, was acquired. Both contact mode and tapping mode measurements gave similar results (Supplementary Fig. 7). The light intensity at the sample location was carefully calibrated using a commercial energy meter (Newport, 91,150 V). Piezoelectric force microscopy was carried out on AR MFP 3D mode under dual AC resonance tracking (DART) mode using a Pt/Ir coated tip with a spring constant of 2 N m^−1^. The imaging voltage *V*_ac_=1 V.

### Photocurrent and ferroelectric polarization measurements

We fabricated a simple device with the two opposite facets of the single crystal coated by semitransparent Pt electrodes and measured the current under illumination through the top electrode. A low-noise probe station and a pA meter/DC voltage source were used and the voltage applied was 1 V. The ferroelectric polarization measurement was conducted using a commercial ferroelectric tester (Radiant Technologies, Precision LC) at different temperatures on a low-temperature probe station.

## Additional information

**How to cite this article:** Zhou, Y. *et al.* Giant photostriction in organic–inorganic lead halide perovskites. *Nat. Commun.* 7:11193 doi: 10.1038/ncomms11193 (2016).

## Supplementary Material

Supplementary Information Supplementary Figures 1-7, Supplementary Notes 1-3 and Supplementary References

## Figures and Tables

**Table 1:** Photostrictive coefficients of different materials.

		**Photostriction,** ***ΔL/L***	**Light intensity,** ***I*** **(W m**^−2^**)**	***(ΔL/L)/I*****(m**^2^** W**^−1^**)**	**Refs**
Non-polar semiconductors	Si crystal	−6.4 × 10^−6^	8.47 × 10^10^†	−7.56 × 10^−17^	26
	Ge crystal	7.84 × 10^−10^	1,000*	7.84 × 10^−13^	27
Polar semiconductors	CdS crystal	7.5 × 10^−5^	1,000*	7.5 × 10^−8^	24,28
	GaAs crystal	4 × 10^−7^	1,000*	4 × 10^−10^	29
Ferroelectric materials	SbSI crystal	4 × 10^−5^	1,000*	4 × 10^−8^	30
	La doped Pb(Zr_*x*_Ti_1−*x*_)O_3_ ceramics	10^−4^	150	6.67 × 10^−7^	31
	BiFeO_3_ crystal	3 × 10^−5^	326	9.2 × 10^−8^	32
	BiFeO_3_ film (35 nm)	4.6 × 10^−3^	∼4 × 10^14^†	1.15 × 10^−17^	33
	PbTiO_3_ film (20 nm)	2.5 × 10^−3^	∼10^15^†	2.5 × 10^−18^	34
Chalcogenide glasses	As_40_Se_25_S_25_Ge_10_ film	4.5 × 10^−4^	1,000*	4.5 × 10^−7^	35
	As_2_Se_3_ film	6.4 × 10^−2^	400	1.6 × 10^−4^	36
	As_2_S_3_ film	5.4 × 10^−2^	400	1.35 × 10^−4^	36
	GeSe_2_ film	−5.6 × 10^−2^	400	−1.4 × 10^−4^	36
	GeS_2_ film	−1.1 × 10^−1^	400	−2.75 × 10^−4^	36
Organic materials	Poly-(4,4′-diaminoazoben-zenepyromelliti-mide) films	−1.2 × 10^−2^	1,000*	−1.2 × 10^−5^	37
	Nematic elastomers	2 × 10^−1^	1,000*	2 × 10^−4^	38
	Poly(ethylacrylate) networks with azo-aromatic crosslinks	2.5 × 10^−3^	1,000*	2.5 × 10^−6^	39
	Diarylethenes	−7 × 10^−2^	5,200	−1.35 × 10^−5^	40
Hybrid perovskites	MAPbI_3_ crystal	5 × 10^−5^	1,000	5 × 10^−8^	Current work
	MAPbI_3_ film (4 μm)	1.25 × 10^−3^	1,000	1.25 × 10^−6^	

^*^The light intensity was not reported in the references and we use 1,000 W m^−2^ (1 Sun) to calculate the photostrictive coefficients.

^†^The light sources were high-energy laser pulses.
