# Supplementary material for: Giant photostriction in organic–inorganic lead halide perovskites
Source: Nat Commun. 2016 Apr 5;7:11193. doi: 10.1038/ncomms11193 (PMC4822042; doi:10.1038/ncomms11193)
Supplement: Supplementary Information — Supplementary Figures 1-7, Supplementary Notes 1-3 and Supplementary References [file ncomms11193-s1.pdf]

a

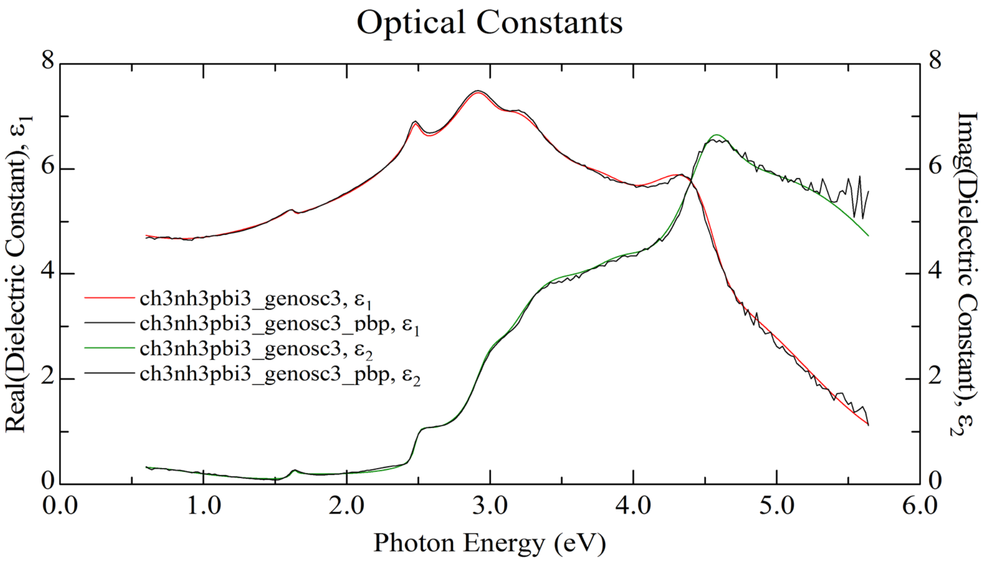

b

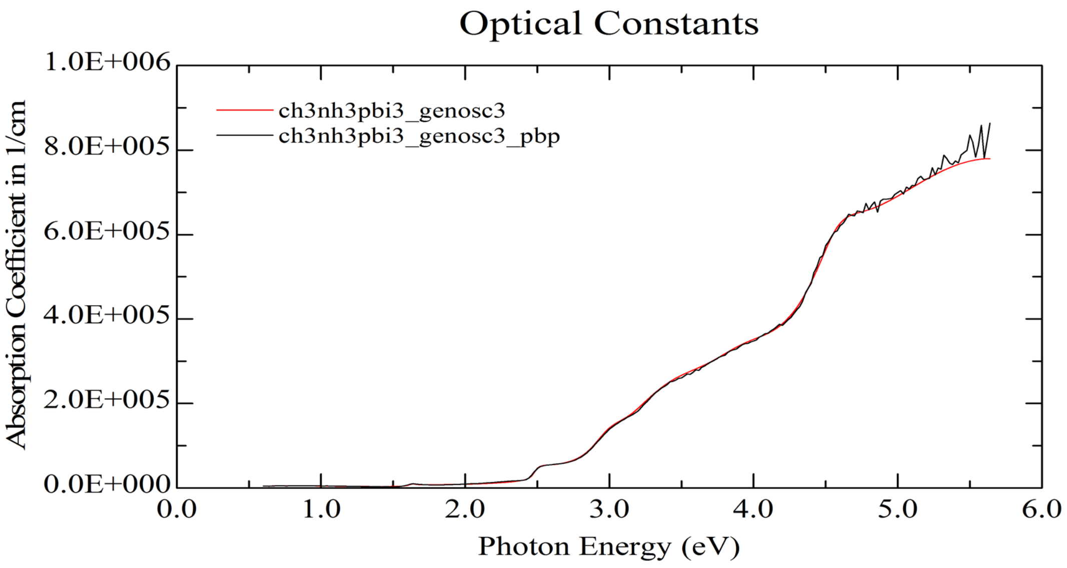

Supplementary Figure 1 | Optical constants of the $MAPbI_3$ crystal deduced from the spectroscopic ellipsometry measurement. (**a**) Real and imaginary parts of the optical dielectric constants of the $MAPbI_3$ single crystal at room temperature. (**b**) Calculated absorption coefficient.

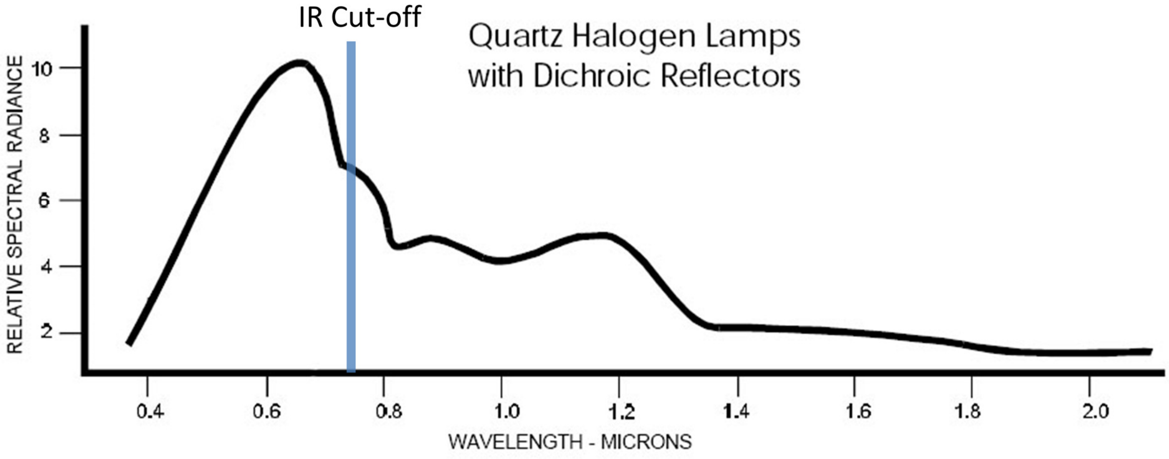

Supplementary Figure 2 | Spectrum of the halogen lamp used in the photostriction test.

Image courtesy of Dolan-Jenner Industries (http://www.dolan-jenner.com/Index.htm).

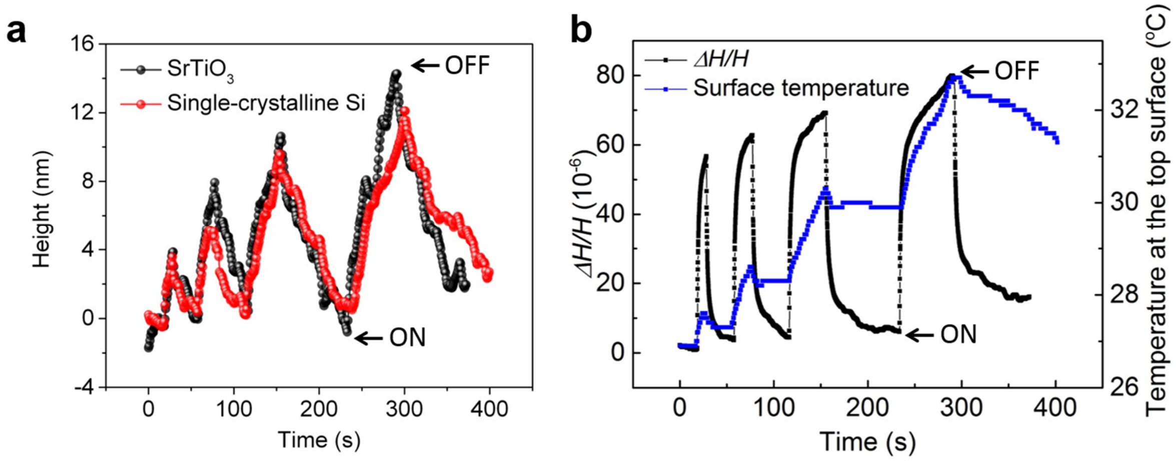

Supplementary Figure 3 | Illumination induced thermal effect. (a) Photo-induced height changes of $SrTiO_3$ and Si single crystals under the same illumination conditions as Fig. 2b (100 mW/cm^2 white light). (**b**) The photostriction and the temperature change in the $MAPbI_3$ single crystal under illumination. Note that no detectable temperature change is observed on the opposite surface of the crystal during the whole period.

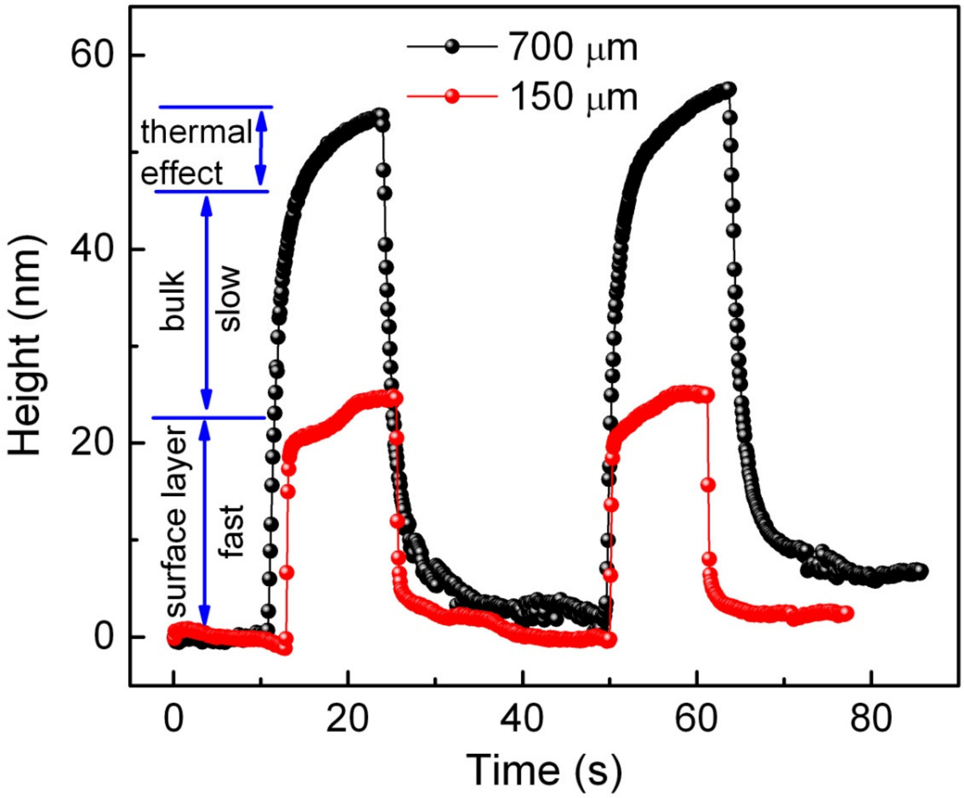

Supplementary Figure 4 | Photostrictive responses of single-crystalline $MAPbI_3$ with different thicknesses.

1. Macroscopic polarization measurement

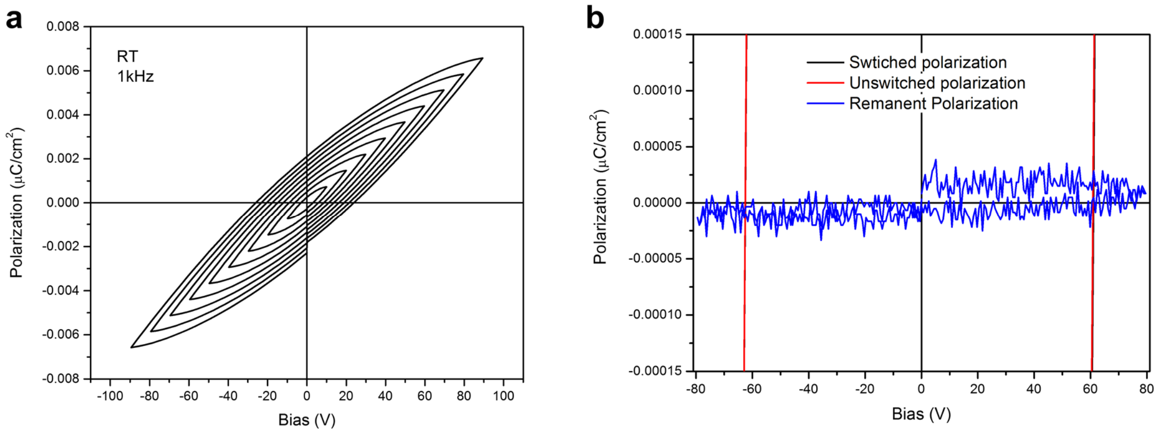

Supplementary Figure 5 | Polarization measurement of the $MAPbI_3$ crystal. (a) Polarization-voltage hysteresis loop exhibits a typical shape of a lossy capacitor with the polarization contribution from both the conductivity and dielectric response of the crystal. (**b)** Remanent-polarization measurement after subtracting the unswitched polarization contribution results in basically zero remanent polarization. Similar behaviors are found for low temperature measurements down to 80 K.

2. Piezoelectric force microscopy measurement

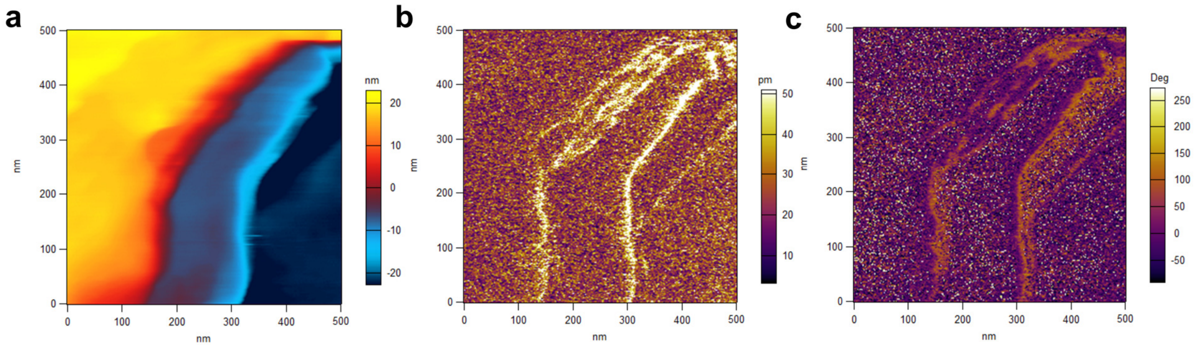

Supplementary Figure 6 | Piezoelectric force microscopy measurement of the cleaved $MAPbI_3$ crystal. (a) Topography, (**b),** Piezoresponse amplitude, and (**c)** Piezoresponse phase images of a freshly-cleaved $MAPbI_3$ crystal on silicon substrate. Despite the topographic artifact at the step edges, no indications of the ferroelectricity can be observed: (1) the amplitude image shows very low response; (2) no domain contrast can be seen in the phase image, (3) no well-defined domain pattern can be formed after poling.

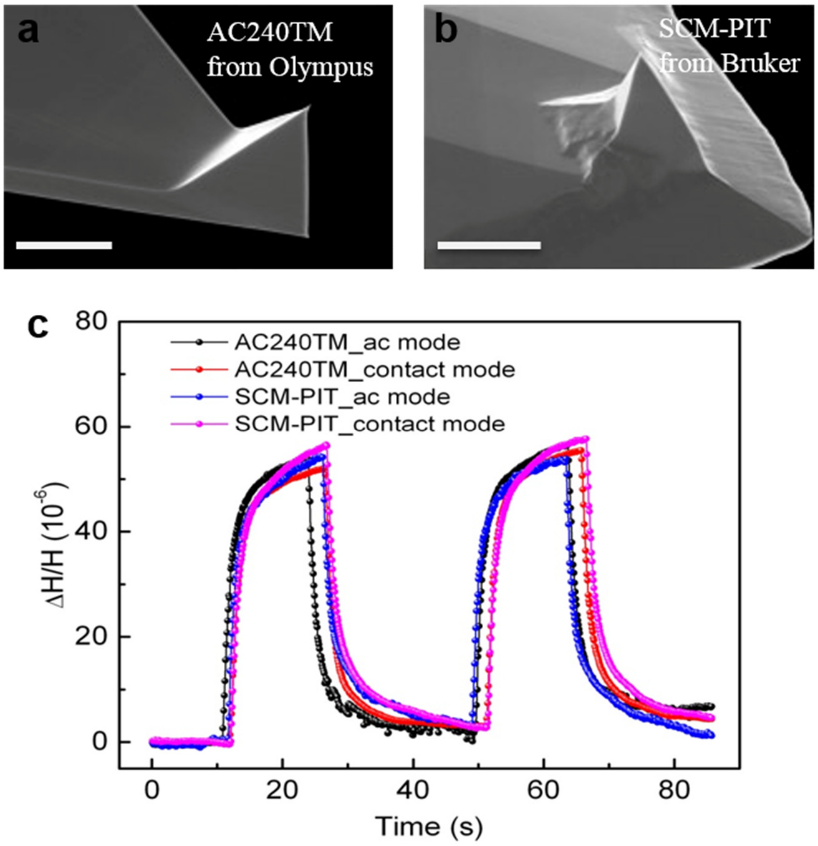

Supplementary Figure 7 | Comparative studies using different AFM tips and under different imaging modes. SEM images of the AFM tip **(a)** AC240TM and **(b)** SCM-PIT used in this study. The scale bars in both images are 10 μm. Image courtesy of Oxford Instrument Asylum Research AFMs. (http://www.asylumresearch.com/Probe/) **(c)** Photostriction of the same $MAPbI_3$ crystal measured using different tips under different imaging modes.

Supplementary Note 1

Ruling out possible experimental artifacts

To exclude any artifacts due to thermal effect or equipment factors, we have carried out careful control tests as well as temperature measurements. As shown in Supplementary Fig. 3a, we tested the height changes of both $SrTiO_3$ and Si single crystals under the same illumination condition and protocol as that for $MAPbI_3$ (Fig. 2b). A few conclusions can be drawn from the observations. First, despite the dramatically different optical absorptions and thermal expansion coefficients, both materials show similar response of height change, indicating this effect is probably material-independent. Second, the response is much slower compared to that observed in $MAPbI_3$, and shows no saturation within illumination time. Third, the maximum response scales with illumination time, suggesting possibly an effect due to heat accumulation. Based on these observations, we infer that this material-independent response is likely from the AFM tip rather than the samples under test. Given that the light is also irradiating the AFM tip, the heating will cause the cantilever to deflect, leading to an artifact of height change. This conclusion is supported by the fact that the deflection of the cantilever is very sensitive to the change of temperature, and it is fully consistent with the slow response and scaling with illumination time.

Furthermore, we have monitored the temperature change of the sample during the photostriction measurement as shown in Supplementary Fig. 3b. The heating rate, however, doesn't correlate with the fast component of height change, but to some extent, agrees with the slow component which we suggest is due to the thermal effect of the AFM tip. Based on the temperature profile, we have performed a simple estimation as follows. Within the photostriction response time of ~1 second, the temperature increase has an upper bound of 0.1 °C. Considering that the thermal

expansion coefficient on the order of 10^{-4}/°C[1,2], the light absorption depth is less than 10 μm and the thermal conductivity is extremely low[3], we can estimate the thermal expansion of ~0.1 nm for $MAPbI_3$, orders of magnitude smaller than the photostriction we observed (~50 nm for a 1-mm-thick crystal). The surface temperature of the silicon single crystal increases by around 1 °C upon 100 mW/cm^2 white light illumination for 1 minute. The smaller temperature change is probably due to the much higher thermal conductivity of silicon (130 W/m·K) compared to $MAPbI_3$ (0.5 W/m·K), which leads to faster heat dissipation. However, even if we assume the temperature of the whole Si single crystal (500 μm thick) increases by 1 °C, given the thermal expansion coefficient of 2.6*10^{-6} /K, the height change is about 1 nm, much smaller than the slow component of the height change on the order of 10 nm. For $SrTiO_3$, due to the negligible absorption in the visible light range, the temperature increase and consequent thermal expansion are even less. Overall, all of the samples show negligible thermal expansion effect, which also supports that the slow response is due to the AFM tip rather than the sample itself.

Moreover, the laser experiment results (Fig. 3b) show that the thermal effect is less for longer light wavelength. In fact, we have measured the surface temperature changes of $MAPbI_3$ under different laser illumination. After illuminated by lasers of the same intensity for 1 minute, the temperature increases on the sample surface are: 4.0 °C (460 nm), 3.1 °C (650 nm), 2.4 °C (808 nm) and 0.4 °C (980 nm), respectively. The difference in the heating rate could be attributed to the dramatically different absorption depths, and thereby the material volumes being heated. We believe that the same reason may be applied to the thermal effect on the AFM tip, which shows different response under illumination with different lasers.

Supplementary Note 2

Thickness-dependence of the photostriction of $MAPbI_3$ single crystal

To fully deconvolute different contributions in the photostrictive response, we performed tests on single crystals with different thicknesses. The flakes were cleaved from the same crystal to eliminate any possible variations among samples. As shown in Fig. S3, for flakes thicker than 700 µm, a saturated response of ~50 nm can be obtained (see Fig. 2b for a 1-mm-thick sample). However, for a 150-µm-thick flake (the thinnest flake we can obtain), the fast photostrictive response dramatically reduces to ~20 nm. This thickness is smaller than the carrier diffusion length estimated for single crystal[4]. Therefore, we argue that although the light absorption depth is only a few micrometers, the photo-excited carriers can diffuse deep into the bulk, which leads to a much thicker responsive layer under illumination. The argument is further supported by the response times of the two samples. The initial 20-nm height increase is fast for both samples, suggesting surface layer contribution. However, the rest contribution in the thicker sample gradually slows down, consistent with a diffusion-limited process. Finally, the plateau due to the thermal effect of the AFM tip appears. The normalized photostriction of the 150-µm-thick flake is above 100 ppm, still much smaller than that of the 4-µm-thick film, because it is not sufficiently illuminated.

Supplementary Note 3

Comparisons between different AFM tips and imaging modes

Since the light in this study is not collimated, the shadowing effect by the AFM tip should be minimal. We have tested the effect of tip shadowing by varying the laser spot size, and observed

no obvious changes in the photostriction. The smallest laser spot size is around 1.7mm^2, while the size of the AC240 cantilever is 30*240 μm^2, about 0.4 % of that of the laser spot. So the shadowing effect is insignificant. To confirm that the photostriction is not affected by the possible shadowing effect, we performed tests using two types of tips with different tip apex locations as shown below. One is at the very end of the cantilever, and hence less shadowing is expected if any. The other one has tip apex completely underneath the cantilever. However, both tips produce identical photostrictive response under the same illumination condition, suggesting that the photostriction is not a localized effect but uniform within the illuminated area. Furthermore, both tapping and contact modes lead to similar results.

Supplementary References

1. Baikie, T. et al. Synthesis and crystal chemistry of the hybrid perovskite (CH3NH3)PbI3 for solid-state sensitised solar cell applications. *J. Mater. Chem. A* **1**, 5628-5641 (2013).
2. Brivio, F. et al. Lattice dynamics and vibrational spectra of the orthorhombic, tetragonal, and cubic phases of methylammonium lead iodide. *Phys. Rev. B* **92**, 144308 (2015).
3. Pisoni, A. et al. Ultra-Low Thermal Conductivity in Organic–Inorganic Hybrid Perovskite CH3NH3PbI3. *J. Phys. Chem. Lett.* **5**, 2488-2492 (2014).
4. Dong, Q.F. et al. Electron-hole diffusion lengths > 175 μm in solution-grown $CH_3NH_3PbI_3$ single crystals. *Science* **347**, 967-970 (2015).
